# Comment from the Editor of the Special Issue: “Lung Disease on COPD, Asthma, Bronchiectasis, Lung Cancer Screening, IPF”

**DOI:** 10.3390/jcm8122060

**Published:** 2019-11-23

**Authors:** Nazia Chaudhuri

**Affiliations:** North West Lung Centre, Manchester University NHS Foundation Trust, Manchester Academic Health Science Centre, Manchester M6 8HD, UK; nazia.chaudhuri@nhs.net

**Keywords:** lung disease, respiratory, pulmonary fibrosis, COPD, asthma, treatment, management

## Abstract

This Special Issue on lung diseases is aimed at giving emergent researchers and clinicians an important forum to share their original research and expert reviews on key topics within respiratory diseases. This Special Issue will be of interest to general physicians and respiratory specialist and will equip the reader with up-to-date knowledge on a wide array of lung diseases, including interstitial lung diseases, COPD, and Asthma.

Lung diseases are amongst the leading causes of mortality worldwide. One-sixth of total deaths are attributed to lung disease, accounting for over 9.5 million deaths worldwide. This burden of disease is the prime reason for this timely Special Issue on lung diseases in the *Journal of Clinical Medicine*. Our objectives are to showcase emergent researchers working at the cutting edge of scientific and clinical advances in the broad field of respiratory diseases.

Here, we provide the reader with comprehensive reviews that would interest those with general medical and specialist respiratory interests. We have two reviews covering chronic obstructive pulmonary disease (COPD), the leading cause of respiratory mortality worldwide. Dey et al. [1] discuss the importance of the imbalance between the protease/antiprotease ratio in the pathophysiology of COPD and the potential of protease directed drugs for the management of COPD, and Trinkmann et al. [2] discuss the emerging concept of the cardiopulmonary continuum, where both cardiovascular and respiratory disease are centrally linked to systemic inflammation. The authors present a comprehensive review of cardiovascular comorbidities in COPD and potential considerations for management of these often coexistent diseases. Shifting the theme from COPD our authors, Or Kalchiem-Dekel et al. [3] from Baltimore, US, describe a practical approach targeted at equipping the general physician with the knowledge and skills to diagnose common interstitial lung diseases (ILDs), with a focus on a comprehensive detailed medical history to delineate the aetiology of ILDs as well as supplementary bloods and imaging. Barratt et al. [4] from the United Kingdom continue this theme, giving the readers a comprehensive overview of the most common and devastating ILD, idiopathic pulmonary fibrosis (IPF). The authors provide the readers with an update on the current insight into pathogenesis of IPF and the clinical trial data regarding the current licenced antifibrotic therapies for IPF—a major paradigm shift in the management of IPF in the last decade. The authors also provide insights into the future emerging therapies for IPF. Furthermore, a potential increasing cause of ILD due to the development of newer biologic and chemotherapeutic agents is presented in this systemic review by Skeoch et al. [5] on drug-induced ILD (DI-ILD). The authors evaluate the current emerging literature on DI-ILD to highlight the common culprits that can cause DI-ILD (cancer drugs, rheumatological drugs, amiodarone, and antibiotics). They discuss the radiological patterns, diagnosis, and management of DI-ILDS. Looking toward future advances in therapy, Fujita et al. [6] present a review of the clinical application of mesenchymal stem cell derived extracellular vesicle-based therapies in lung disease.

This Special Issue on lung diseases also presents a number of primary research articles spanning all aspects of respiratory diseases showcasing emergent researchers at the forefront of advances in respiratory medicine. We present a variety of themes from novel aspects of lung physiology testing in pulmonary hypertension to the prevalence of fatigue in asthmatic patients and the use of macrolide therapies during hospitalisation of children with childhood disease, amongst other interesting primary research articles, which would be of interest to our growing number of readers of the *Journal of Clinical Medicine*.
